# Saving threatened plant species: Reintroduction of Hill’s thistle (*Cirsium hillii*. (Canby) Fernald) to its natural habitat

**DOI:** 10.1371/journal.pone.0231741

**Published:** 2020-04-16

**Authors:** Bita Sheikholeslami, Mukund Shukla, Christina Turi, Cavan Harpur, Praveen K. Saxena

**Affiliations:** 1 Department of Plant Agriculture, Gosling Research Institute for Plant Preservation, University of Guelph, Guelph, ON, Canada; 2 Bruce Peninsula National Park, Tobermory, ON, Canada; National University of Kaohsiung, TAIWAN

## Abstract

Hill’s thistle (*Cirsium hillii* (Canby) Fernald) is a perennial plant endemic to the Great Lakes region of North America. Hill’s thistle is listed as threatened in Ontario and Canada where it is found in globally rare alvar habitats. The main objective of this study was *ex-situ* conservation of Hill’s thistle using *in vitro* culture techniques and reintroduction of micropropagated plants back to their natural habitat in Bruce Peninsula National Park, Ontario, Canada. Two out of twenty-nine available seeds were successfully germinated under *in vitro* condition. An efficient micropropagation protocol was optimized with 100% survival during acclimatization of plantlets in the greenhouse. Three hundred micropropagated plants were reintroduced to twelve different sites within Bruce Peninsula National Park in June and July 2017. Plants were monitored for survival, rosette growth, and flowering on all sites from 2017–2019. After four months of planting, 67 to 99% of the plants were alive in different sites and 90 to 99% of them survived over winter. In the following years, shoot regeneration and flowering were observed on most sites. This study further confirms the benefit of plant tissue culture techniques to ensure revival of Hill’s thistle ecological biodiversity through the reintroduction of micropropagated plants. This approach consisting of the components of conservation, propagation, and reintroduction (CPR) may potentially serve as a model for saving and enriching other species at risk.

## Introduction

According to the Committee on the Status of Endangered Wildlife in Canada (COSEWIC), there are seven hundred and forty-eight wildlife species at risk in Canada, with one hundred and ninety-six species belonging to the vascular plant taxon [1]. One of these species is Hill’s thistle (*Cirsium hillii*. (Canby) Fernald), a perennial thistle with spiny, shallow lobed, basal leaves [2]. Flowering of Hill’s thistle plants occurs from mid-June to August and the floral stem consists of a flower head on one or multiple branches with a cluster of pinkish-purple flowers [3]. Hill’s thistle flowers support many pollinators including bumble bees, small carpenter bees, sweat bees, leaf cutter bees, and brush-footed butterflies [4]. An important pollinator is *Bombus pensylvanicus*, also a threatened bee species native to North America [3]. In the spring, it is also a food source for local herbivores [5].

Hill’s thistle is listed as threatened on the Species at Risk in Ontario (SARO) list under the Endangered Species Act (ESA) and under Schedule 1 of the Species at Risk Act (SARA), and is ranked globally, nationally and sub-nationally as vulnerable [5]. In the U.S.A., it is listed nationally as vulnerable, and critically imperiled in Illinois, Indiana and Iowa [3]. In Canada, Hill’s thistle populations are restricted to Southern Ontario where they are found on 93 sites localized to four areas including Bruce County, Simcoe County, the Manitoulin District and the surrounding islands [5]. Hill’s thistle is found in various vegetation types, as classified by Lee et al. [6] in the Ecological Land Classification of Ontario (ELC), such as open alvar, shrub alvar, treed alvar, tallgrass woodland, open sand barren, coniferous forest and tallgrass prairie [5]. Hill’s thistle populations in Ontario are often associated with open habitat on shallow soils over limestone bedrocks, such as alvars [3]. Alvars are open dry habitats with a little to no canopy cover and are subjected to extreme environmental conditions such as drought, flood, ice and natural fires [3]. Ecological succession as a result of fire suppression, has led to the accumulation of organic matter on the ground resulting in a transition from an open area to mixed-forest growth [7]. Manual disturbance in these habitats can act as a substitute for fire, clearing the forest vegetation and improving vascular plant diversity in unburned alvar woodlands [8]. In addition to open alvar, Hill’s thistle plants have also been observed growing in areas with disturbance such as hiking trails and roadsides [5].

Loss of suitable habitat is the primary threat to Hill’s thistle in Canada [3,5]. Alvars are globally rare and are threatened in Ontario by quarrying, shoreline development, recreational use and ecological succession due to fire suppression [3,9]. White-tailed deer are also a potential threat as extensive damage from grazing has been observed on Manitoulin Island [5]. Reintroduction is a tool used to revive extirpated populations within their indigenous range [10]. Conventional methods of plant reintroductions often involve sowing seeds directly *in situ* or transplanting germinated seedlings such as with reintroduced *Cirsium pitcheri* (Torr. ex Eat.) Torr. & A. Gray [11,12]. However, low flowering was reported for *C*. *pitcheri* in transplanted seedlings and for another reintroduced species *Arnica montana* L., survival after several years was low [12,13]. This may be due to slow growth resulting in longer times needed to achieve maturity. Low seed germination rates have been reported in greenhouse studies which is common with native thistles [4,5] and this may be a threat to this and many other species. An additional limitation is the restriction for collecting large quantities of material from Schedule 1 species at risk in Canada as it can negatively impact extant populations [5]. In 2004, COSEWIC [3] reported fewer than 500 mature flowering Hill’s thistle individuals present in Canada, however this number is now estimated to be closer to 1000 mature individuals [5]. Hill’s thistle requires cross-fertilization and is at risk for inbreeding depression, however, high genetic diversity was determined in eleven populations in Ontario compared to other congeneric rare species *C*. *pitcheri* with habitat loss as the primary concern for conservation efforts [14].

The impact of extirpation and extinction of plant biodiversity due to habitat loss [9] may be reduced and managed by replenishing the declining extant populations. The combination of conservation practices and biotechnology is currently being researched for commercially and ecologically important species at risk in several countries. Micropropagation, an advanced plant tissue culture technique, is a tool that can be used to maintain living germplasm and produce large quantities of plants in a controlled environment from a limited starting material to provide plants for conservation [15]. Micropropagation is emerging as an efficient tool that has been successfully used as a source of healthy plant material for reintroductions globally [15–17]. In Canada, micropropagation has also been applied to propagate plants for *in vitro* conservation of endangered plant species such as *Betula lenta* L. and *Castilleja levisecta* Greenm [18,19]. Micropropagation is a good approach for Hill’s thistle because germplasm can be stored to conserve the limited genetic diversity in sterile conditions while the threats to the extant populations are managed through reintroduction of micropropagated plants. *In vitro* cultured tissues remain viable for extended periods of time and are more reliable than seed storage where seed viability is reduced in low temperatures over time [20]. The main objective of this study was to evaluate the potential of the conservation, propagation, and reintroduction (CPR) model for Hill’s thistle recovery and enrichment *in situ* through reintroduction of micropropagated plants.

## Materials and methods

### Culture initiation

Seeds were received from Michael Patrikeev, Bruce Peninsula National Park, Parks Canada Agency, Tobermory, ON (Permit no. BPF-2015-19858) in July 2015. Seeds were surface sterilized for ten minutes in a 10% (v/v) bleach solution (Clorox©, The Clorox company; 5.4% sodium hypochlorite) containing ca. 0.01% (v/v) Tween-20® (Sigma-Aldrich, Missouri, USA) and rinsed thrice with sterile deionized water for three minutes each wash. The seeds were then placed in sterile polystyrene disposable Petri dishes (VWR CATALYST Laboratory Services, Pennsylvania, USA) with semi-solid medium containing Murashige and Skoog (MS) [21] basal salts (PhytoTechnology Laboratories, Kansas, USA), 1 mL L^-1^ Gamborg’s B5 [22] vitamins (PhytoTechnology Laboratories), 3% sucrose and 2 mL L^-1^ Plant Preservative Mixture (PPM; Plant Cell Technology, ON, Canada). Phytagel ^TM^ (Sigma-Aldrich, Canada) was added at 2.2 g L^-1^ after the pH was adjusted to 5.7 with 1 M sodium hydroxide or 1 M hydrochloric acid (Fisher Scientific Company, Ontario, Canada). Medium was autoclaved for twenty minutes at 121°C and 118 kPa and allowed to cool in sterile conditions.

Later, the seeds were kept in the dark for seven days and then transferred to the tissue culture growth room at a temperature of 25°C on shelves with two fluorescent bulbs (Osram Sylvania Ltd., Mississauga ON) that emit 40 μmol m^-2^ s^-1^ in a 16 h light/8 h dark cycle. Individuals derived from these seedlings were labelled as lines HT1, HT2 and HT3 in order to indicate their different parental origin. The labelled seedlings were transferred to medium consisting of the same components as above with the addition of 2.2 μM 6-benzylaminopurine (Phytotechnology Laboratories, Kansas) to establish multiple cultures.

### Shoot multiplication

The effects of four cytokinins were tested on shoot tips to optimize *in vitro* multiplication in order to develop the highest number of shoots in fourteen days. Four-week old shoot tips were transferred to Petri dishes containing MS basal medium supplemented with 6-benzylaminopurine (BA), 2-isopentenyladenine (2-IP), zeatin (ZEA) or kinetin (KIN) at 0, 1, 2, 5 or 10 μM. Preliminary experiments noted stunted growth when concentrations of BA exceeded 10 μM in the medium (S1 Fig) and hence 10 μM was chosen as the highest concentration (S1 Fig). The culture medium also consisted of MS basal salts, 1 ml L^-1^ Gamborg’s B5 vitamins, 3% sucrose, 2.2 g L^-1^ Phytagel^TM^ and pH was adjusted to 5.7. Petri dishes were kept in the tissue culture growth room. The number of shoots was recorded after fourteen days as preliminary experiments determined that shoots proliferated within two weeks of culture.

### Rooting and acclimatization

The effects of two auxins were tested on four-week old shoot tips with two to three intact leaves to optimize *in vitro* root induction. Auxin, 1-Naphthaleneacetic acid (NAA) or Indole-3-butyric acid (IBA) at 0, 5, 10 or 20 μM was added to the medium that consisted of MS basal salts, 1 ml L^-1^ Gamborg’s B5 vitamins, 3% sucrose and 2.2 g L^-1^ Phytagel^TM^ with pH adjusted to 5.7. The number of roots was recorded after thirty-five days.

To determine survival in the greenhouse, rooted plantlets from semi-solid medium were rinsed with deionized water to remove any excess medium and then transferred to 18-cell trays containing soil mix, Sunshine® Mix #4 (Sun Gro Horticulture Canada Ltd., Vancouver, Canada). Trays were placed in the mist bed for five days and then transferred to greenhouse benches where watering occurred once every three days. The greenhouse compartment was programmed to have a constant temperature of 23°C during the day and 18°C at night with a photoperiod of 16 h, and a light intensity of 250 μmol m^-2^ s^-1^. The number of days in the mist bed were selected from preliminary experiments which showed high survival rate after five days. Survival rate was recorded fifteen days after the plantlets had been transferred to the greenhouse bench. Our routine transplant experiments showed that the plantlet that survived in the first two weeks, remained alive and continued to grow in the greenhouse conditions.

### Transplant design for reintroduction of plants

**Fig 1:**
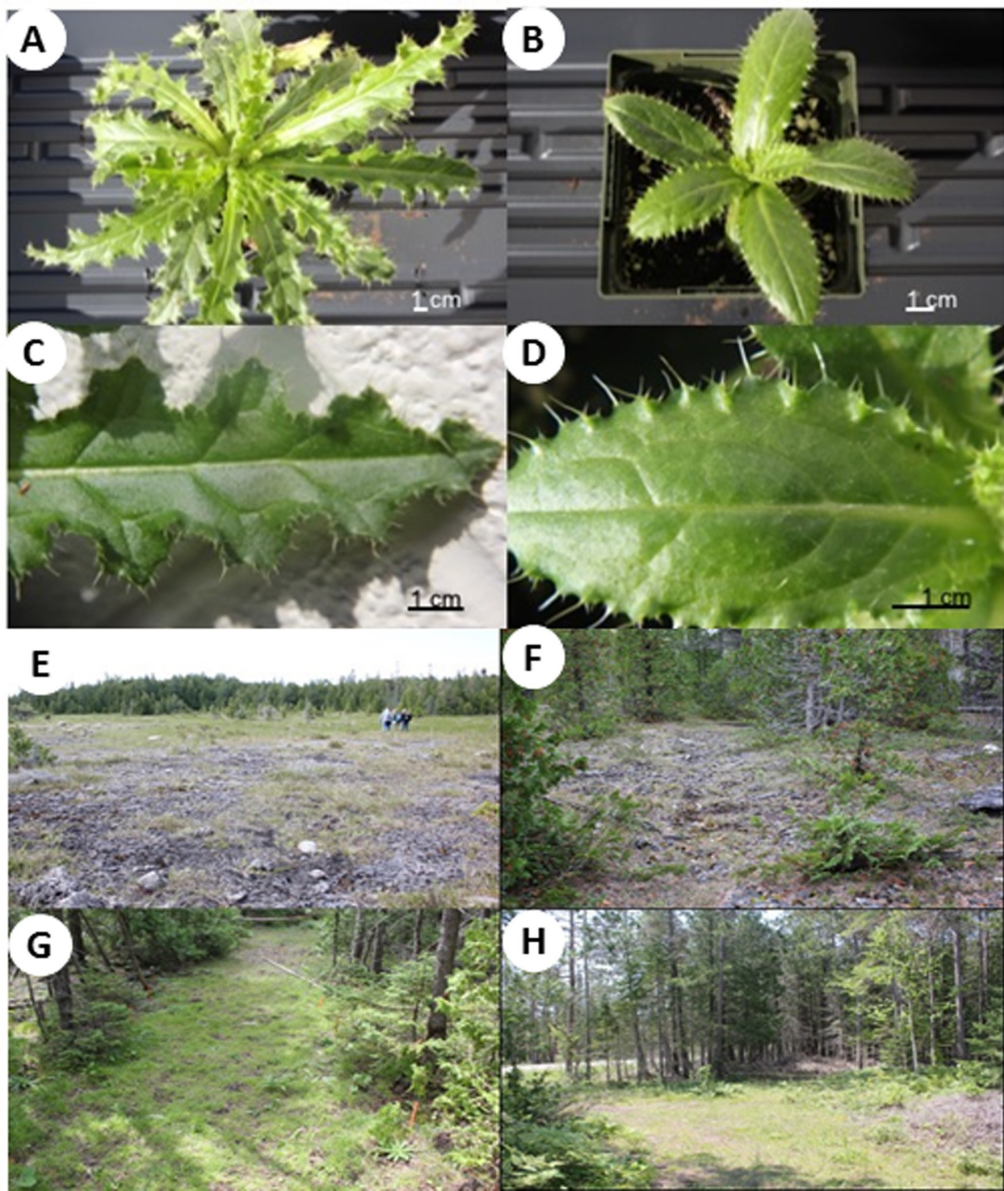
Micropropagated Hill's thistle plants grown in the greenhouse for (A) twenty-five days and (B) fifteen days. Close up images show the effects of age on leaf spine development for plants grown for (C) twenty-five days and (D) fifteen days. Images of the four types sites where micropropagated Hill’s thistle plants were reintroduced in Bruce Peninsula National Park in Tobermory, Ontario (open alvar (E), shrubbed alvar (F), treed alvar (G) and non-alvar (H)).

Three hundred plants from lines HT1 and HT3 were multiplied from shoot cultures and rooted in Plant Growth Regulator (PGR) free, MS basal medium for twenty-one days. Plantlets were then transferred to soil (Sunshine Mix #4; Sun Gro Horticulture Canada Ltd., Vancouver, Canada) pots in the mist bed for five days. After the mist bed plants to be used in the first reintroduction transplant were grown on the greenhouse bench for twenty-five days (Fig 1A) whereas plants for the second transplant were grown for fifteen days (Fig 1D). Leaves from the first transplant were considerably damaged during the transport to Bruce Peninsula National Park, therefore the plants used in the second transplant were grown for shorter time to have smaller rosettes (Fig 1A–1D). For further hardening, all plants were placed in a vinyl dome enclosure for twenty-four hours before transporting and kept on their respective sites for twenty-four hours before transplanting.

### Site selection

Hill’s thistle plants were transplanted on two separate days (June 2 and July 18, 2017) in twelve sites within Bruce Peninsula National Park in Tobermory, Ontario (45.26 N, 81.66W) which is located 300 kilometres north of Toronto, Ontario. The Bruce Peninsula National Park was established in 1987 to protect diverse ecosystems and plant biodiversity. The park is the core protected area of the UNESCO Niagara Escarpment World Biosphere Reserve. We received the permit (BPF-2015-19858) from Michael Patrikeev, Bruce Peninsula National Park, Parks Canada Agency, 7374 Highway # 6, Tobermory, Ontario to collect the plant materials, conduct field plantation of Hill’s thistle and gather field observations. One hundred and fifty plants were transplanted on each date to three alvar sites: open alvar, shrubbed alvar, treed alvar and three non-alvar sites (Table 1 and Fig 1E–1H). Twenty-five plants, sixteen from line HT1 and nine from line HT3, were randomly assigned to plots within an area of 20 m^2^ on each site. Sites were selected according to canopy cover by the surrounding vegetation.

**Table 1:** Description of sites used to reintroduce micropropagated Hill’s thistle plants in June 2, 2017 (site 1 to 6) and July 18, 2017 (site 7 to 12) at Bruce Peninsula National Park in Tobermory, Ontario. Twenty-five plants were randomly assigned to plots within an area of 20 m^2^ on each site.

Site #	GPS Latitude	GPS Longitude	Site	Alvar Type	Habitat Type
1	45.12	-81.54	Huron Road 1	Open	Grassy field on a property next to Huron Road.
2	45.19	-81.60	Pendall Point 1	Shrub	Shrubbed area next to open alvar North West of Dorcas Bay.
3	45.18	-81.58	Side Creek 1	Treed	Hiking Trail off Dorcas Bay Road on the South East of Dorcas Bay.
4	45.16	-81.58	Cecil Watson	Non	Trail with motor vehicle tracks off Dorcas Bay Road.
5	45.13	81.54	Johnsons 1	Non	Grassy area covered in Trees on the side of Johnsons Harbour Road.
6	45.19	-81.58	Singing Sands Lot	Non	Treed area next to Singing Sands Parking Lot on the South East of Dorcas Bay Road.
7	45.12	-81.54	Huron Road 2	Open	Grassy area further into the alvar site Huron Road 1.
8	45.19	-81.60	Pendall Point 2	Shrub	Area further into alvar site Pendall Point 1.
9	45.18	-81.58	Side Creek 2	Treed	Area further into the trail after alvar site Side Creek 1.
10	45.19	-81.62	Sand Dune	Non	Open sand dune next to forested area on Eagle Road.
11	45.13	81.54	Johnsons 2	Non	Trail off the side of Johnsons Harbour Road, Hiking Trail in a forested area.
12	45.15	-81.46	Hay Field	Non	Hay field overgrown with various vegetation off Hidden Valley Road.

### Site characteristics

Soil pH was measured to determine uniformity of the twelve sites. Three soil samples were collected at each site to measure soil pH with a portable Exstik PH100 pH meter (EXTECH Instruments, Massachusetts, USA). Each site had three replicates randomly chosen from soil dug up from plots at the time of the transplants and three measurements were taken from each sample.

### Introduction of micropropagated plants

Survival rates for both transplants were recorded in fall 2017 to determine the success of introducing micropropagated plants into natural habitats. Plants were also monitored for survival, growth, and flowering at all sites during the years 2018 and 2019. Observations for survival rates were recorded after winter in the month of May 2018 and May 2019. After the full development of flowers, observations for the occurrence flowering were recorded in July 2018 and July 2019. Plants were scored as alive, dead or eaten by herbivores based on the state of their leaves: rosettes with green leaves were considered alive, rosettes with complete brown leaves as dead and plants with no shoot tissue left were recorded as eaten. The absence of green shoot tissue but roots present in the soil were taken as the signs of complete herbivory. Plants with signs of grazing by insects or animals that did not consume the entire shoot were considered alive. Herbivory was compared among sites used only in the second transplant.

Rosette diameters of micropropagated plants and natural plants were measured from the first transplant. Rosettes significantly affected by herbivore grazing in the second transplant were, were not assessed and included in the analysis. Photos of plants on sites 1 to 6 were taken at two-month intervals on June 27, August 9 and October 10. Rosette diameter was calculated by taking three measurements from each rosette with ImageJ 1.x Software [23]. To determine the effect of the site on rosettes, diameters of micropropagated plants were compared between the sites for each date. To determine the difference in growth between micropropagated plants and natural plants, diameters of micropropagated plants were compared to five natural plants on each site, with the exception of site 2 where no such plants were present.

### Overwinter survivorship

Overwinter survival rates and regeneration rates were recorded during the year 2018 and 2019. Surviving plants were evaluated for three stages of growth and development: a vegetative rosette, a cluster of regenerated shoots and a flowering plant. Plants with no rosette or rosettes with brown leaves were considered dead. The effect of site on flowering of plants was assessed to determine the ideal sites for growth of the transplants.

### Statistical analyses

Data was analyzed using a one-way ANOVA with PROC GLIMMIX in SAS 9.4 software (SAS Institute Inc. Cary, North Carolina, USA). The *in vitro* experiments consisted of ten Petri dishes with three shoot tips in each dish. All experiments were repeated twice. Normality was tested using Shapiro-Wilk’s test of normality. For all responses, the normal distribution and constant variance assumptions on the error terms were verified by examining the residuals. When the effects were significant, means were compared using Tukey-Kramer Honest Significant Difference (HSD) test with an alpha value of 0.05. Data represents mean ± standard error from 25 plants per site. Graphical and or tabular form of results are presented for ease of understanding.

## Results

### Culture initiation

**Fig 2:**
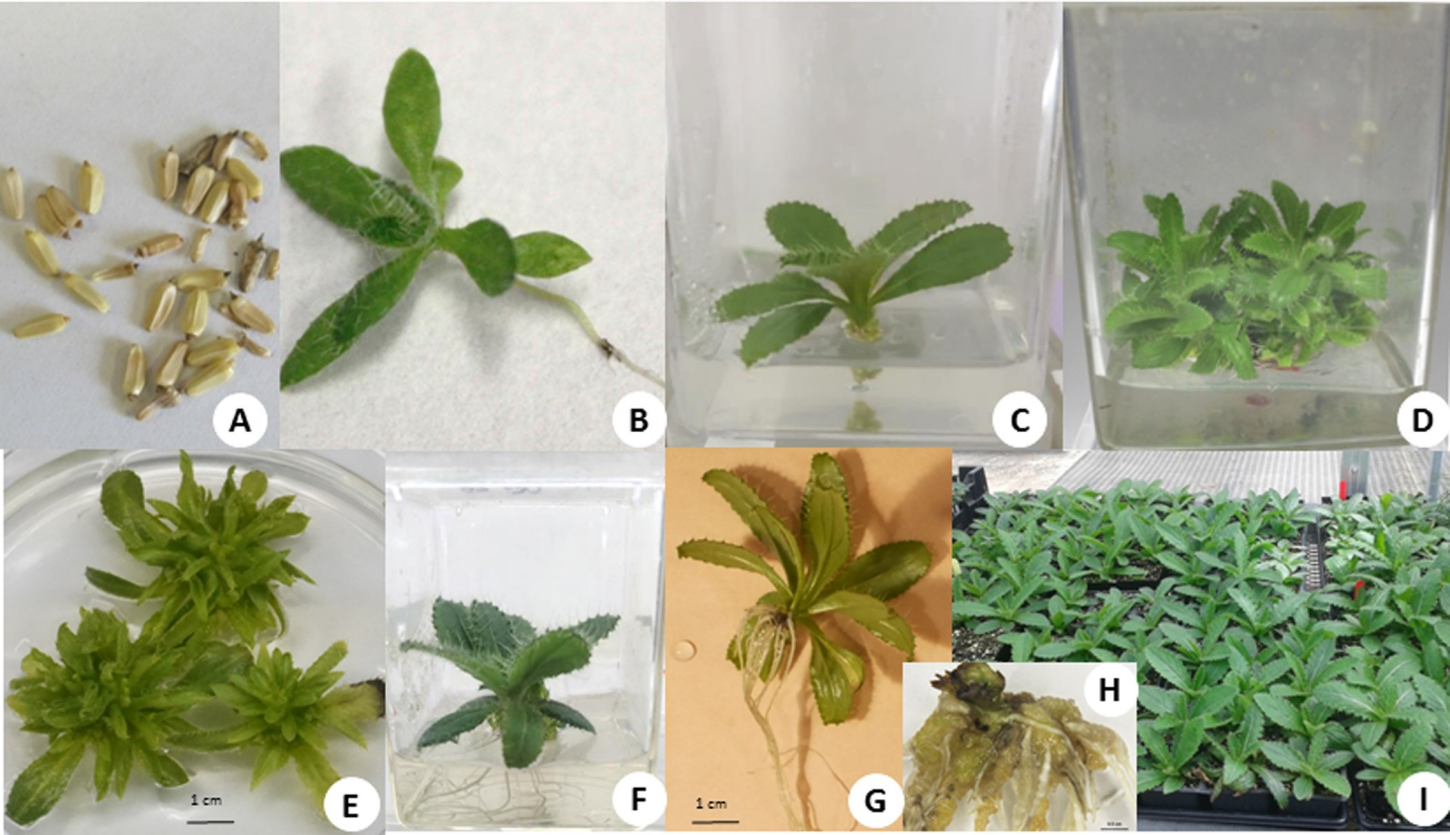
Hill’s thistle Seeds (A) were collected from Parks Canada, Tobermory and germinated under *in vitro* conditions (B). Three weeks old *in vitro* shoots (C) were subcultured on the shoot multiplication medium with BA at 5.0 μM (D) and 10.0 μM (E) to multiply shoots. *In vitro* rooting observed when individual shoots were subcultured on the medium without auxin (F), with NAA at 5.0 μM (G) and 20.0 μM NAA (H). All rooted plants were acclimatized in the greenhouse conditions (I) before transplanting to their natural habitat.

Three seeds germinated from twenty-nine seeds received from Bruce Peninsula National Park. The seedling labelled HT2 died during culture *in vitro*, thus, lines were developed from seedlings labeled HT1 and HT3 (Fig 2A–2C).

### Shoot multiplication

**Fig 3:**
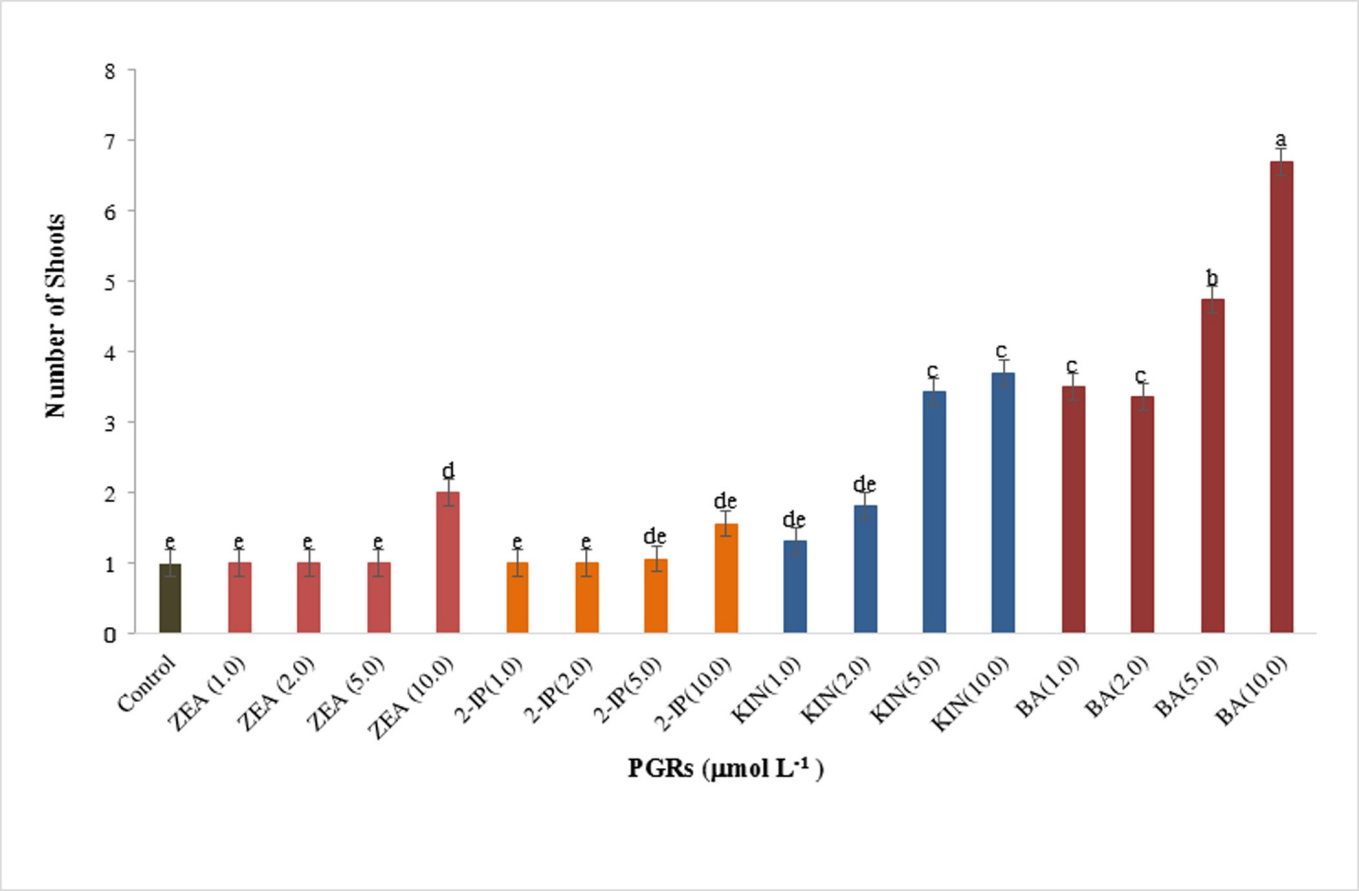
The effects of four cytokinins, zeatin (ZEA), 2-isopentenyladenine (2-IP), kinetin (KIN) and 6-benzylaminopurine (BA) on numbers of shoot after 5 weeks of culture. Bars represent means ± standard error, where means followed by the different letters are significantly different according to Tukey-Kramer HSD test. Each level consisted of five biological replicates.

Multiple shoots were produced in response to all concentrations of BA tested, at 5 μM and 10 μM of KIN, and at 10 μM of ZEA (Figs 3 and S2). Shoot tips did not multiply in the medium with 2-IP and 1 μM, 2 μM and 5 μM of ZEA. In the absence of PGRs, shoot tips grew as a single rosette and each shoot developed roots (S2 Fig). Roots were not present on the single rosettes in response to cytokinin treatments. The highest number of shoots was observed in the treatment with 10 μM BA (6.71 shoots) followed by 5 μM BA (4.75 shoots, Fig 2D), however, the shoots were stunted with smaller leaves at 10 μM (Figs 2E and S2). KIN at 5 μM and 10 μM produced similar numbers of shoots as BA at 1 μM and 2 μM. At 10 μM ZEA, shoots proliferated but were significantly lower in number than those with the other levels where prolific multiplication was observed. The medium supplemented with BA at 5 μM was considered optimal for shoot multiplication.

### Rooting and acclimatization

Root induction was observed in all treatments with NAA and IBA (Fig 2F, 2G and 2H). However, the addition of NAA to the culture medium significantly increased the number of roots whereas cultures with IBA were similar to the control (S3 Fig). Roots were observed after seven days in the treatment with IBA at 5 μM and the control (Fig 2F), whereas roots were observed after twenty-one days in NAA and IBA at 20 μM. The highest numbers of roots were produced with NAA at 10 μM (7.15 roots per shoot) followed by 5 μM (6.11 roots per shoot, Fig 2G) and 20 μM (5.81 roots per shoot). The number of roots was not significantly different between the NAA treatments (S3 Fig). However, callus formation was observed in NAA treatments at 10 μM and 20 μM (Fig 2H) except NAA at 5 μM (Fig 2G). Roots from NAA at 10 μM and 20 μM were sensitive to breaking off during the transfer from semi-solid medium to soil in the greenhouse. The highest number of roots with no callus was produced in the treatment with NAA at 5 μM. Callus formation was not observed in the IBA treatments as well as control. Nevertheless, all plantlets from different treatments including the control survived on the greenhouse benches following a five-day period in the mist bed (Fig 2I). Normal shoot and root development was observed without callus formation in the control treatment, and hence was considered as an optimal medium for *in vitro* rooting.

### Site characteristics

The soil pH was relatively uniform ranging between 7.4 and 8.3. The highest soil pH was observed on sites 3, 4, 5 and 10 with the lowest on site 12. Open alvar sites, 1 and 7, and shrub alvar sites 2 and 8 showed similar pH values. However, treed site 3 had a significantly higher soil pH than site 9. The soil pH of non-alvar sites varied between the sites from approximately pH 7.4 to 8.3.

### Reintroduction of micropropagated plants

**Fig 4:**
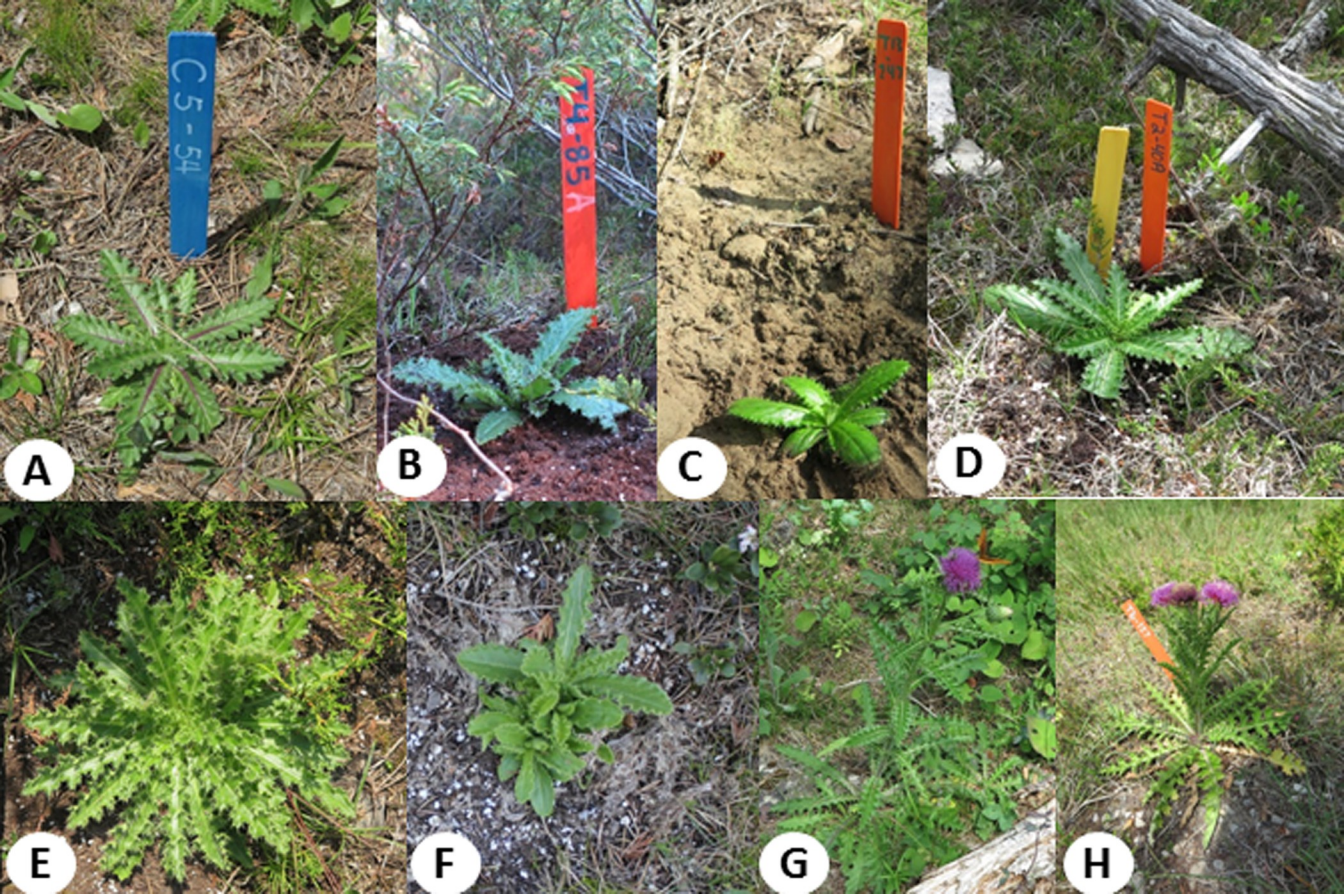
Natural sites of Hill’s thistle were selected where natural population existed (A). Micropropagated Hill's thistle plants were transplanted on June 2017 (B) and July 2017 (C) on 12 different sites at the National Park in Tobermory, ON, Canada. Micropropagated plant growth and development were observed after one month and three months of transplanting (D and E). Multiple shoots were observed after winter survival of micropropagated Hill's thistle plants in May 2018 (F). Flowering with well-formed flowerheads observed in the natural (G) and micropropagated plants (H) in July 2018 with multiple flowerheads on micropropagated plants (H).

Survival was high in the first transplant (99%), with 100% survival on the treed alvar and all three non-alvar sites with natural plant populations (Fig 4A). There was no difference in survival between the two lines (HT1 and HT3) for both transplants (Table 2 and Fig 4B). One plant died on shrub site 2 with a brown rosette and one plant had been eaten on site 1. In the second transplant (Fig 4C), herbivory affected overall survival (67%). Herbivory was observed on open, shrub and treed alvar sites and no herbivory was observed on the non-alvar sites (Table 2). Signs of grazing were observed on most plants on the alvar sites; however, these plants had green and intact shoot apices and were considered alive. Complete herbivory was highest on treed alvar site 9 (80%) and was significantly higher than that on open site 7 (60%) or on shrub site 8 (52%).

**Table 2:** Survival rates and herbivory rates recorded in October 2017 of micropropagated plants reintroduced in the twelve sites at Bruce Peninsula National Park, Tobermory, Ontario. The first planting in June occurred on sites 1 to 6 and the second planting in July occurred on sites 7 to 12. Twenty-five plants were randomly assigned to plots within an area of 20 m^2^ on each site. Data represents mean ± standard error from 25 plants per site. Means followed by different letters in columns are significantly different according to Tukey’s HSD test (*P*-value < 0.05).

Site Type		Name	Transplant Survival Rate (%)	Herbivory Rate (%)
First Planting				
Open alvar	1	Huron Road 1	96 ± 3.9a	4 ± 3.9
Shrub alvar	2	Pendall Point 1	96 ± 3.9a	0.00
Tree alvar	3	Side Road Alvar 1	100 ± 0a	0.00
Non alvar	4	Cecil Watson	100 ± 0a	0.00
Non alvar	5	Johnson Harbour 1	100 ± 0a	0.00
Non alvar	6	Singing Sands	100 ± 0a	0.00
Second Planting				
Open alvar	7	Huron Road 2	40 ± 9.8bc	60 ± 9.8ab
Shrub alvar	8	Pendall Point 2	48 ± 10.0bc	52 ± 10.0ab
Tree alvar	9	Side Road Alvar 2	20 ± 8.0c	80 ± 8.0a
Non alvar	10	Sand Dune	96 ± 3.9a	0.00b
Non alvar	11	Johnson Harbour 2	100 ± 0a	0.00b
Non alvar	12	Hay Field	96 ± 3.9a	0.00b

Herbivory rate was compared among sites used only in the second planting and hence letter grouping was provided.

One month after the first transplant in June 2017, rosette diameters remained similar to the time of transplanting on all sites except site 6 (Fig 4D), where the plants were significantly larger (28.4 cm). In August, the significantly larger plants were observed on site 6 and site 2 compared to the other sites. In October, rosette diameters were smaller than previous months from leaf die-back, however site 2 and site 6 still had the largest plants (Fig 4E), whereas site 4 and site 5 had the smallest plants.

In October 2017, micropropagated plants were found to be significantly larger than natural plants on site 1 (*P* = 0.0043) and were similar to the natural plants on the other sites (Fig 4A) where natural plants were present. The natural plant sizes varied among the six sites with the largest plants on site 6 and the smallest plants on site 1.

### Overwinter survivorship

High overwinter survival was observed for reintroduced Hill’s thistle plants as a single rosette, regenerated shoots or a flowering plant (Table 3 and Fig 4F and 4H). Of the plants that were alive in October 2017, the rate of overwintering was 99% for those from the first transplant, and 90% for those from the second transplant. Completely eaten plants in October had 0% survival in May 2018. Overall, for all reintroduced plants, 98% of those from the first transplant and 60% of those from the second transplant were alive in the following year. Adventitious shoot regeneration was observed in May 2018 on all alvar sites and on sites 4, 5, and 11 (Fig 4F). The shrub alvar sites 2 and 10 had high shoot regeneration rates (40%) followed by open alvar sites 1 and 8 (16%). Shoot regeneration was not observed on plots where complete herbivory occurred in 2017. In July 2018, flowering was observed on all sites except for sites 8 and 10, with the highest occurrences on sites 1 (80%) and 6 (80%). A similar trend in plant survival was observed in May 2019, with a range of 74–90% of plants surviving, only one of the twelve sites showed a lower (52%) survival rate. Moreover, the micropropagated plants were found to grow with multiple shoots in the range of 1–5 shoots from the same original location (Fig 4F). Flowering was observed on natural plants (Fig 4G) as well as on micropropagated plants (Fig 4H) in the month of July 2018 and there was a site to site variation in the number of flowers on each plant (Fig 4H). The rate of flowering varied from 20 to 80% amongst all the different sites of first planting (Table 3). However, very limited flowering (<10%) was observed on natural plants.

**Table 3:** The percentages of plants showing overwinter survival and shoot regeneration recorded in May 2018 and flowering in July 2018 of all micropropagated Hill’s thistle plants reintroduced to Bruce Peninsula National Park in Tobermory, Ontario. The first planting in June 2017 occurred on sites 1 to 6 and the second planting in July 2017 occurred on sites 7 to 12. Twenty-five plants were randomly assigned to plots within an area of 20 m^2^ on each site. Data represents mean ± standard error from 25 plants per site. Means followed by different letters in columns are significantly different according to Tukey’s HSD test (P-value < 0.05).

Site Type		Name	Overwinter Survival (%)	Flowering (%)	Shoot Regeneration (%)
First Planting					
Open alvar	1	Huron Road 1	96 ± 3.9a	80 ± 8.1a	16 ± 7.3a
Shrub alvar	2	Pendall Point 1	96 ± 3.9a	44 ± 9.8ab	40 ± 9.8a
Tree alvar	3	Side Road Alvar 1	100 ± 0a	44 ± 9.8ab	4 ± 3.9a
Non alvar	4	Cecil Watson	100 ± 0a	20 ± 8.1b	8 ± 5.4a
Non alvar	5	Johnson Harbour 1	96 ± 3.9a	36 ± 10.1b	8 ± 5.4a
Non alvar	6	Singing Sands	100 ± 0a	80 ± 8.1a	0.00a
Second Planting					
Open alvar	7	Huron Road 2	40 ± 10.3bc	4 ± 3.9	16 ± 7.3
Shrub alvar	8	Pendall Point 2	40 ± 10.3bc	0.00	40 ± 9.8
Tree alvar	9	Side Road Alvar 2	20 ± 8.0c	4 ± 3.9	8 ± 5.4
Non alvar	10	Sand Dune	92 ± 5.2a	0.00	0.00
Non alvar	11	Johnson Harbour 2	92 ± 5.2a	4 ± 3.9	8 ± 5.4
Non alvar	12	Hay Field	76 ± 8.7ab	16 ± 7.3	0.00

For the first planting, over winter survival percentage, flowering and shoot regeneration were compared among sites and for second planting only overwinter survival percentage was compared. Hence, a letter grouping was provided for those means.

## Discussion

Plant conservation strategies are of utmost importance to prevent population decline and maintain biodiversity. Micropropagation, an application of plant tissue culture technique for mass propagation of plants, holds tremendous potential to benefit plant conservation by producing healthy plants and conserving germplasm lines from limited source material in a controlled environment. Furthermore, micropropagated plants generated from limited starting material can be used as a source to replenish declining populations, to reintroduce individuals to areas with extirpated populations, and to study habitat suitability for reintroduction of new plant populations. The major goal of our research is to preserve threatened and endangered plant biodiversity through the application of *in vitro* culture technologies that can be used to prevent species loss in the field. Earlier we developed micropropagation methods for several threatened North American plant species including American elm, cherry birch, and golden paintbrush [18,19,24,25]. The current study referred to as the CPR model was undertaken to assess the potential of micropropagated plants in establishing new plant populations in a range of diverse natural habitats. We selected Hill’s thistle, ranked as a threatened or vulnerable plant species, globally, nationally and sub-nationally [5] for reintroduction in natural habitat of the species. Hill’s thistle is of ecological importance as it supports life cycle of many bee pollinators including *B*. *pensylvanicus*, a threatened bee species native to North America [3,4] and also serves as a food source for local herbivores [5]. Existing methods to improve the status of Hill’s thistle in Southern Ontario involve maintaining the extant populations and protecting the rare alvar habitats where they are found [5]. In addition to restriction on seed collection, low numbers of flowering plants and low seed germination rates also limit the efficacy of conventional methods of plant conservation for recovery of Hill’s thistle. Our results of the CPR trial conducted with Hill’s thistle in the Bruce Peninsula National Park, Tobermory, Ontario, confirm that micropropagation technologies can be successfully applied in the propagation of threatened species and enrich their populations *in situ*.

Hill’s thistle seeds, collected by Parks Canada from natural population in the park in fall of 2015, were used to develop micropropagation protocol for propagating plants for *ex situ* conservation. Low germination rates as reported for Hill’s thistle seeds in the greenhouse conditions [3] were also observed in this study. Besides low viability, seeds may not have germinated from a lack of appropriate environmental signals or physical factors such as scarification to break dormancy [26]. However, two seedlings recovered from the 29 seeds available were sufficient to initiate *in vitro* shoot cultures.

PGRs are commonly added to the culture medium to induce shoot proliferation from meristematic cells. Cytokinins and auxins used independently or in combinations are known to regulate organ development and are inducers of cell division, shoot initiation and multiplication [27–30]. The cytokinin used for shoot multiplication needs to be optimized for each species and the concentration can be genotype-specific [31]. BA was the best cytokinin for Hill’s thistle and has been found to be effective for other endangered plants including *Ficus Carica* L., *B*. *lenta*, *C*. *levisecta*, *Rhaponticoides mykalea* (Hub.Mor.) M.V. Agab & Greuter and *Isoplexis isabiliana* (Webb & Berth) Masf. [18,19,32–34]. Stunted shoots at high concentrations of BA have also been observed in other plant species [35]. With a high concentration of ZEA, shoot proliferation was observed, however, it was not as effective for shoot proliferation in Hill’s thistle as it was in other thistle species such as *Silybum marianum* (L.) Gaertn [36]. The shoots when grown on the medium with an added exogenous auxin (NAA or IBA) led to root induction and whole plantlet development. In this study, NAA had a positive effect on root number, which is similar to its effect in *R*. *mykalea* and *Primula heterochroma* Stapf. [34,37]. A lower response of root induction was also observed in PGR-free media similar to that observed for *S*. *marianum* [36]. Higher number of roots on Hill’s thistle plantlets had no effect on survival as all of the plantlets acclimatized after five days in the mist bed survived. However, high numbers of roots may have beneficial long-term effects such as improved growth *in situ* particularly in alvar sites that are rocky and have nutritionally poor soil.

Plantlets in the mist bed for nine days had 100% survival. Mist beds which have high humidity have also been observed to benefit plantlet growth in *Cirsium arvense* plants that grew 80% more shoot dry weight in high relative humidity than in low relative humidity [38]. The mist bed is necessary to reduce the shock of transferring plants from nearly 100% humidity in the culture vessels to the greenhouse and subsequently *in situ*. The integrated approach applied to optimize the micropropgation protocol for shoot multiplication involved supplementing the medium with BA [5 μM), allowing root development in the basal medium and acclimatizing rooted plants in the mist bed for two weeks before transferring them to the greenhouse in order to achieve nearly 100% survival rate.

The survival and growth of micropropagated Hill’s thistle plants *in situ* were affected by unique biotic and abiotic factors found on each site in Bruce Peninsula National Park. The size of the plant at the time of transplant was an important factor as nearly all the large plants survived from the first transplant. Whereas herbivore grazing reduced survival in the second transplant as Hill’s thistle plants with smaller leaves were more vulnerable to herbivory and none of the plants that were completely eaten survived in the following year (Table 2). Treed site 9 had the highest occurrences of herbivory, although site 3 and site 9 were on the same hiking path, complete herbivory was not observed on site 3 with the larger rosettes. The opposite was observed in studies with *C*. *pitcheri*, where herbivory was observed in larger rosettes compared to smaller ones [39]. With *C*. *pitcheri*, leaf damage was caused by insects that consumed segments of the leaves. Daws and Koch (2015) determined that herbivory can be reduced by barricading the reintroduction sites [40]. Physical barriers may improve survival rates of smaller Hill’s thistle plants, however complete herbivory could be reduced in future transplants by taking advantage of the species natural defence system, spiny leaves, which seems to be a more effective deterrent as seen with larger thistle plants. Plant and herbivore interactions are common in nature and shape plant defense systems [41] depending on the morphological and phytochemical characteristics of the species and access to herbivores. Herbivory was also a limiting factor in reintroduction studies with micropropagated plants of *Cattleya intermedia* and *Mammillaria mathildae* Kraehenbuehl & Krainz [42]. However, herbivory was found to have a positive effect on survival of *C*. *intermedia* [43]. Plants that were grazed on by herbivores generated new roots, shoots and leaves changing the susceptibility to herbivory. Thus the effect of herbivory may be plant specific as well as determined by the stage of development and should be investigated in long-term studies of species recovery.

This study determined that although all of the selected sites are potential habitats for enriching Hill’s thistle populations, certain sites are more suitable than others. For example, the results indicated that site 1 had the optimal conditions for growth and flowering even though the natural plants in the site were small. This may be due to the soil characteristics, moisture content and temperature variations among sites. Soil moisture may also have an effect on Hill’s thistle plants as the largest plants on sites 2 and 6 were closer to Dorcas Bay which is in close proximity of Lake Huron compared to the other sites. Pence et al. (2011) also observed that *Minuartia cumberlandensis* (B.E. Wofford & Kral) McNeill plants thrived in areas with moderate levels of light and soil moisture [17]. While light and soil moisture influenced plant growth, the survival of reintroduced plants of seven different genetic lines of *M*. *cumberlandensis* was not significantly affected [17]. In our study also the genetic lines showed no difference in survival as both lines HT1 and HT3 had similar survival in the greenhouse and the fields. Also, Hill’s thistle micropropagated plants were similar in size to natural plants on sites 3, 4, 5 and 6 and larger than the natural plants on site 1. This is a reflection of the robust nature and ability of plants in adaptation to local environment. Micropropagated plants may accumulate significantly lower biomass than the wild plants depending on the species and conditions of transplant and growth as observed by Juliani et al. (2011) for reintroduced *Lippia junelliana* (Moldenke) Tronc. plants [16].

Micropropagated plants showed high survival rate after the winter season, but the overwinter period negatively impacted survival especially for smaller plants from the second transplant compared to large plants from the first transplant. Interestingly, in the case of micropropagated plants more than one plant regenerated after both the years of overwintering which indicates the presence of multiple meristems in the rosette or the regeneration of multiple shoots from the root. Multiple shoot regeneration suggests that juvenile nature of micropropagated plants supports vigorous growth following initial establishment of plants in their natural habitat. This observation further highlights the significance of an optimized micropropagation protocol to propagate robust plants. Furthermore, flowering of plants was observed after only one winter season in this study. Higman and Penskar (1996) reported that Hill’s thistle flowering often occurs after three years [44]. The high percentage and continued flowering of micropropagated plants observed following the first transplant and after winter periods over two years further supports the assumption about the role of the juvenile nature of these plants. Flowering is a high metabolic energy driven process, and vigorous growth of plants in the vegetative phase may have contributed to a better flowering response of reintroduced plants. Higher flowering was observed on those sites which are close to water bodies which indicates that soil moisture may also play a role in the induction of flowering. It would be interesting to explore potential role of plant hormones at different stages of growth, adaptations, and flowering responses which are predominantly determined by endogenous profiles of auxin, cytokinins, and other plant growth regulators including indoleamines [45]

In conclusion, the CPR model developed in this study provides evidence that *in vitro* technologies can play an important role in species recovery projects and enhance natural populations in areas suffering from population decline. This model may be useful for saving other species at risk and their reintroduction in natural habitats. The micropropagated Hill’s thistle plants also offer an interesting system to research the mechanisms of survival, adaptation to natural environments, and flowering, which combined with analyses of site-specific influences on plant growth and methods to mitigate herbivory, could further increase plant survival in future transplant efforts.

## Supporting information

S1 Fig An image for shoot multiplication on the medium supplemented with 6-benzylaminopurine (BA) at 10 μM after 5 weeks of *in vitro* shoot culture. (TIF)

S2 Fig A combined representative image for shoot multiplication on the medium supplemented with different cytokinins, zeatin (ZEA), 2-isopentenyladenine (2-IP), kinetin (KIN) and 6-benzylaminopurine (BA) at various levels (1, 2, 5, 10 μM) after 5 weeks of *in vitro* shoot culture. (TIF)

S3 Fig The effects of two auxin, 1-Naphthaleneacetic acid (NAA) or Indole-3-butyric acid (IBA) at 0, 5, 10 or 20 μM on numbers of root after 5 weeks of *in vitro* shoot culture for rooting. Bars represent means ± standard error, where means followed by the different letters are significantly different according to Tukey-Kramer HSD test. Each level consisted of five biological replicates.
(TIF)

## References

[1] COSEWIC. Canadian Wildlife Species at Risk. Committee on the Status of Endangered Wildlife in Canada. [Internet]. 2017. Available from: http://www.registrelep.gc.ca/sar/assessment/wildlife_species_assessed_e.cfm

[2] Moore RJ, Frankton C. An evaluation of the status of *Cirsium pumilum* and *Cirsium hilli*. Can J Bot. 1966;44(5):581–95. 10.1139/b66-070

[3] COSEWIC. COSEWIC assessment and status report on Hill’s thistle Cirsium hillii in Canada. Committee on the Status of Endangered Wildlife in Canada. Ottawa; 2004.

[4] Eckberg J, Lee-Mäder E, Hopwood J, Jordan SF, Brianna B. Native Thistles: A Conservation Practitioner’s Guide Plant Ecology, Seed Production Methods, and Habitat Restoration Opportunities. Portland,OR: The Xerces Society for Invertebrate Conservation; 2017 92 p.

[5] OMNR. Recovery strategy for the Hill’s thistle (Cirsium hillii) in Ontario. Ontario Recovery Strategy Series. Ontario Ministry of Natural Resources, Peterborough, Ontario. Adoption of Recovery Strategy for the Hill’s thistle (Cirsium hillii) in Canada (Parks Ca. Queen’s Printer for Ontario; 2013. 84 p.

[6] Lee HT, Bakowsky WD, Riley J, Bowles J, Puddister M, Uhlig P, et al Ecological land classification for southern Ontario: First approximation and its application. Ontario Minist Nat Resour. 1998;1–225.

[7] Parsons DJ, DeBenedetti SH. Impact of fire suppression on a mixed-conifer forest. For Ecol Manage. 1979;2:21–33. 10.1016/0378-1127(79)90034-3

[8] Catling P. Vascular Plant Diversity in Burned and Unburned Alvar Woodland: More Evidence of the Importance of Disturbance to Biodiversity and Conservation. Can Field-Naturalist. 2009;123(3):240–5. 10.22621/cfn.v123i3.971

[9] Robinson J, Hermanutz L. Evaluating human-disturbed habitats for recovery planning of endangered plants. J Environ Manage. 2015;150:157–63. 10.1016/j.jenvman.2014.10.033 25485935

[10] IUCN/SSC. Guidelines for Reintroductions and Other Conservation Translocations. Version 1.0. Gland, Switzerland: IUCN Species Survival Commission. Ecologial Applications 2013 57 p.

[11] Maunder M. Plant reintroduction: an overview. Biodivers Conserv. 1992;1:51–61. 10.1007/BF00700250

[12] Bell T, Bowles M, McEachern A. Projecting the Success of Plant Population Restoration with Viability Analysis In: Brigham CA, Schwartz MW, editors. Population Viability in Plants. Springer, Berlin, Heidelberg; 2003 p. 313–48.

[13] Godefroid S, Piazza C, Rossi G, Buord S, Stevens AD, Aguraiuja R, et al How successful are plant species reintroductions? Biol Conserv. 2011;144:672–82. 10.1016/j.biocon.2010.10.003

[14] Freeland JR, Gillespie J, Ciotir C, Dorken ME. Conservation genetics of Hill’s thistle (Cirsium hillii). Botany. 2010;88(12):1073–80. 10.1139/B10-080

[15] Loyola-Vargas VM, Ochoa-Alejo N. An introduction to plant cell culture: The future ahead In: Methods in Molecular Biology. 2012 p. 1–8. 10.1007/978-1-61779-818-4_1 22610615

[16] Rodolfo JH, Koroch AR, Zygadlo JA, Trippi VS. Evaluation of micropropagation for the introduction into cultivation and conservation of Lippia junelliana, an endemic aromatic plant from Argentina. Ind Crops Prod. 2011;34:1353–7. 10.1016/j.indcrop.2010.12.009

[17] Pence VC, Plair BL, Charls SM, Clark JR, Taylor DD. Micropropagation, Cryopreservation, and Outplanting of the Cumberland Sandwort Minuartia cumberlandensis. J Ky Acad Sci. 2011;72:91–9. 10.3101/1098-7096-72.2.91

[18] Rathwell R, Shukla MR, Jones AMP, Saxena PK. In vitro propagation of cherry birch (Betula lenta L.). Can J Plant Sci. 2016;96:571–8. 10.1139/cjps-2015-0331

[19] Salama A, Shukla MR, Popova E, Fisk NS, Jones MP, Saxena PK. In vitro propagation and reintroduction of golden paintbrush (Castilleja levisecta), a critically imperilled plant species. Can J Plant Sci. 2017;98:762–70. 10.1139/cjps-2017-0207

[20] Dooley FD, Wyllie-Echeverria S, Van Volkenburgh E. Long-term seed storage and viability of *Zostera marina*. Aquat Bot. 2013 11;111:130–4. ISSN: 0304–3770

[21] Murashige T, Skoog F. A revised medium for rapid growth and bioassays with tobacco tissue cultures. Physiol Plant. 1962;15:473–97. 10.1111/j.1399-3054.1962.tb08052.x

[22] Gamborg OL, Miller RA, Ojima K. Nutrient requirements of suspension cultures of soybean root cells. Exp Cell Res. 1968;50(1):151–8. 10.1016/0014-4827(68)90403-5 5650857

[23] Schneider CA, Rasband WS, Eliceiri KW. NIH Image to ImageJ: 25 years of image analysis. Nat Methods. 2012;9:671–5. 10.1038/nmeth.2089 22930834 PMC5554542

[24] Shukla MR, Jones AMP, Sullivan JA, Liu C, Gosling S, Saxena PK. In vitro conservation of American elm (Ulmus americana): Potential role of auxin metabolism in sustained plant proliferation. Can J For Res. 2012;42(4):686–697. 10.1139/x2012-022

[25] Uchendu E, Paliyath G, Brown DCW, Saxena PK. In vitro propagation of North American ginseng (Panax quinquefolius L.). In Vitro Cell Dev Biol—Plant. 2011;47:710–8. 10.1007/s11627-011-9379-y

[26] Penfield S. Seed dormancy and germination. Curr Biol. 2017;27:R874–909. 10.1016/j.cub.2017.05.050 28898656

[27] Skoog F, Miller CO. Chemical regulation of growth and organ formation in plant tissues cultured *in vitro*. Symp Soc Exp Biol. 1957;11:118–30. 13486467

[28] George EF, Hall MA, Klerk G-J De. Plant Growth Regulators II: Cytokinins, their Analogues and Antagonists In: George EF, Hall MA, Klerk G-J De, editors. Plant Propagation by Tissue Culture: Volume 1 The Background [Internet]. Dordrecht: Springer Netherlands; 2008 p. 205–26. Available from: 10.1007/978-1-4020-5005-3_6

[29] Schaller GE, Bishopp A, Kieber JJ. The yin-yang of hormones: Cytokinin and auxin interactions in plant development. Plant Cell. 2015;27:44–63. 10.1105/tpc.114.133595 25604447 PMC4330578

[30] Chickarmane VS, Gordon SP, Tarr PT, Heisler MG, Meyerowitz EM. Cytokinin signaling as a positional cue for patterning the apical–basal axis of the growing Arabidopsis shoot meristem. Proc Natl Acad Sci. 2012;109(10):4002–7. Available from: http://www.pnas.org/content/109/10/4002.abstract doi: 10.1073/pnas.1200636109 22345559 10.1073/pnas.1200636109 PMC3309735

[31] Pérez-Tornero O, Burgos L, Egea J. Introduction and establishment of apricot *in vitro* through regeneration of shoots from meristem tips. In Vitro Cell Dev Biol—Plant. 1999;35:249–53. 10.1007/s11627-999-0087-9

[32] Arrebola ML, Verpoorte R. Micropropagation of Isoplexis isabelliana (Webb & Berth.) Masf., a threatened medicinal plant. J Herbs, Spices Med Plants. 2003;10:89–94. 10.1300/J044v10n02_10

[33] Shahcheraghi ST, Shekafandeh A. Micropropagation of three endemic and endangered fig (Ficus carica L.) genotypes. Adv Hortic Sci. 2016;30:129–34. https://www.jstor.org/stable/26525345

[34] Hayta S, Bayraktar M, Baykan Erel S, Gurel A. Direct plant regeneration from different explants through micropropagation and determination of secondary metabolites in the critically endangered endemic Rhaponticoides mykalea. Plant Biosyst. 2017; 151:20–28. 10.1080/11263504.2015.1057267

[35] Silva AL, Rogalski M, Guerra MP. Effects of different cytokinins on in vitro multiplication of Prunus ‘Capdeboscq’ rootstock. Crop Breed Appl Biotechnol. 2003;3:149–56. ISSN: 1518–7853

[36] Rady MR, Saker MM, Matter MA. *In vitro* culture, transformation and genetic fidelity of Milk Thistle. J Genet Eng Biotechnol. 2018;2:563–72. 10.1016/j.jgeb.2018.02.007 PMC6353781 30733774

[37] Sharaf ARN, Hamidoghli Y, Zakizadeh H. In vitro seed germination and micropropagation of primrose (Primula heterochroma Stapf.) an endemic endangered Iranian species via shoot tip explants. Hortic Environ Biotechnol. 2011;52:298–302. 10.1007/s13580-011-0129-1

[38] Hunter JH, Hsiao AI, McIntyre GI. Some Effects of Humidity on the Growth and Development of *Cirsium arvense*. Bot Gaz. 1985;146:483–8. 10.1086/337552

[39] Stanforth LM, Louda SM, Bevill RL. Insect herbivory on juveniles of a threatened plant, Cirsium pitcheri, in relation to plant size, density and distribution. Ecoscience. 1997;4:57–66. 10.1080/11956860.1997.11682377

[40] Daws MI, Koch JM. Long-term restoration success of re-sprouter understorey species is facilitated by protection from herbivory and a reduction in competition. Plant Ecol [Internet]. 2015 4;216(4):565–76. Available from: 10.1007/s11258-015-0459-7

[41] Barton KE, Hanley ME. Seedling-herbivore interactions: Insights into plant defence and regeneration patterns. Ann Bot. 2013;112:643–50. 10.1093/aob/mct139 23925939 PMC3736773

[42] García-Rubio O, Malda-Barrera G. Micropropagation and reintroduction of the endemic Mammillaria mathildae (Cactaceae) to its natural habitat. HortScience. 2010;45:934–8. 10.21273/HORTSCI.45.6.934

[43] Endres JD, Sasamori MH, Schmitt JL, Droste A. Survival and development of reintroduced *Cattleya intermedia* plants related to abiotic factors and herbivory at the edge and in the interior of a forest fragment in south Brazil. Acta Bot Brasilica. 2018;32(4):555–6. 10.1590/0102-33062018abb0009

[44] Higman PJ, Penskar MR. Special plant abstract for Cirsium hillii (Hill’s thistle) In Michigan Natural Features Inventory, Lansing, MI; 1996 p. 2.

[45] Erland L, Saxena P. Auxin driven indoleamine biosynthesis and the role of tryptophan as an inductive signal in Hypericum perforatum (L.). PLoS One. 2019;42(10):e0223878 10.1371/journal.pone.0223878 PMC6797091 31622392

