# Supplementary figures and images for: Saving threatened plant species: Reintroduction of Hill’s thistle (Cirsium hillii. (Canby) Fernald) to its natural habitat
Source: PLoS One. 2020 Apr 16;15(4):e0231741. doi: 10.1371/journal.pone.0231741 (PMC7162482; doi:10.1371/journal.pone.0231741)

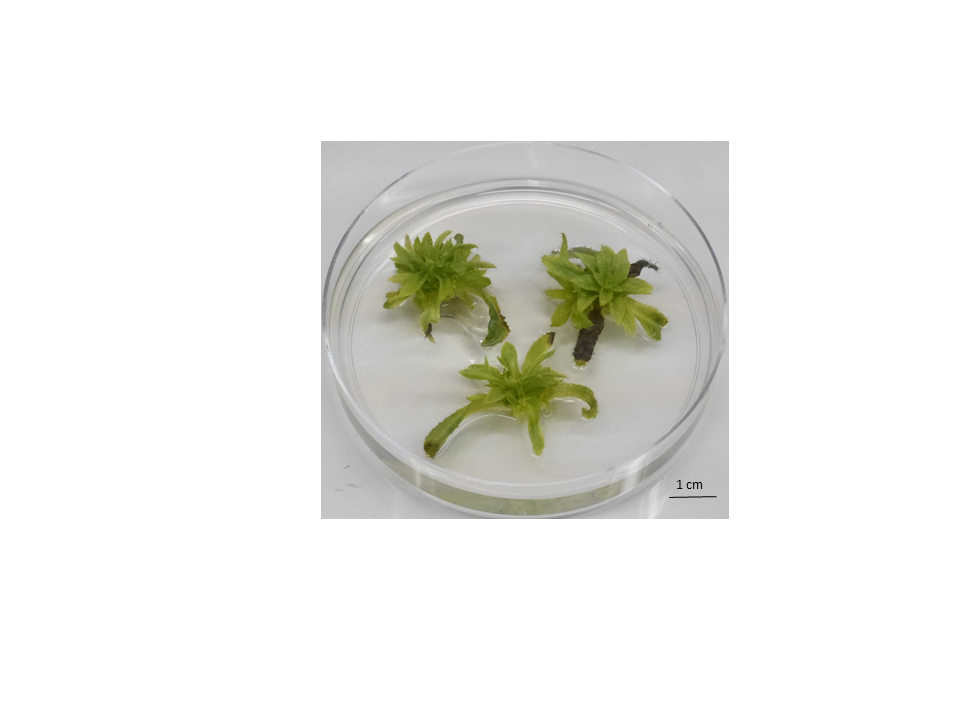

Supplement: S1 Fig — (TIF) [file pone.0231741.s001.tif]

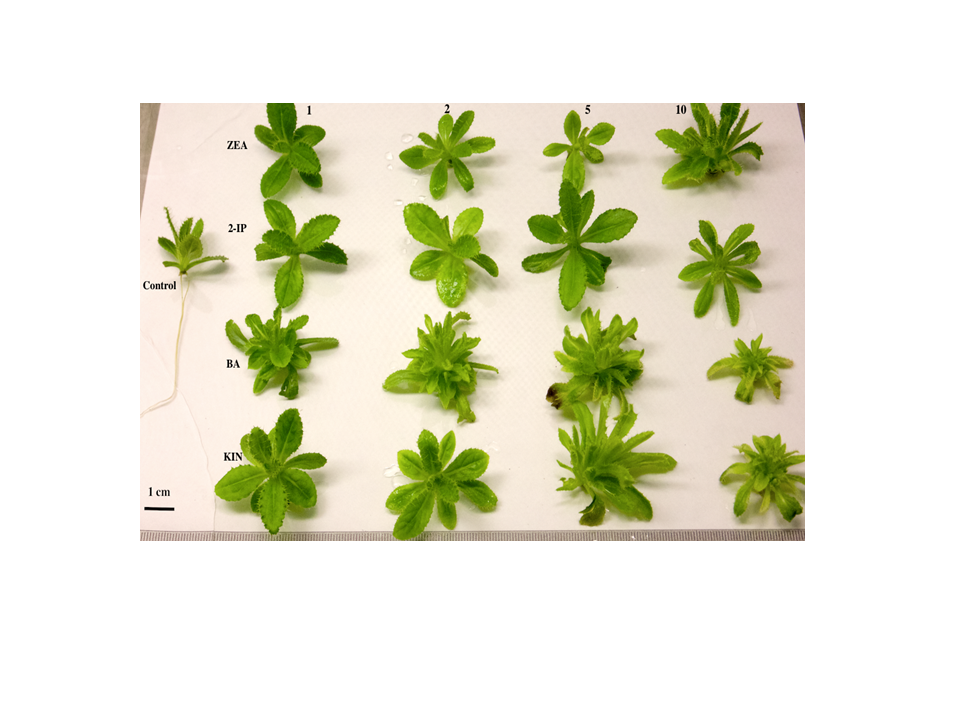

Supplement: S2 Fig — (TIF) [file pone.0231741.s002.tif]

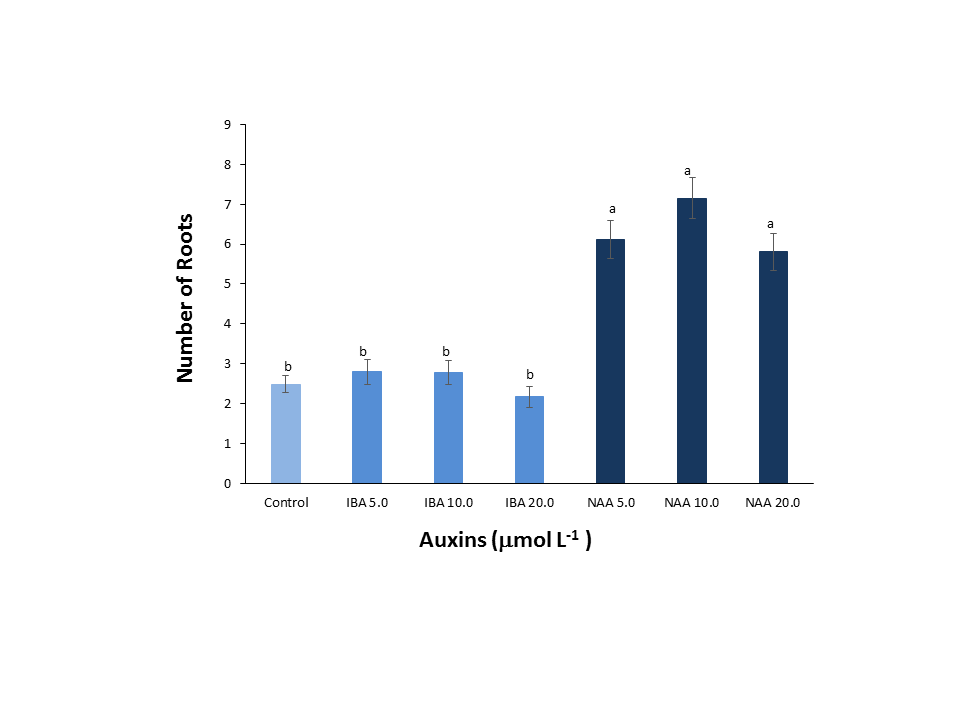

Supplement: S3 Fig — Bars represent means ± standard error, where means followed by the different letters are significantly different according to Tukey-Kramer HSD test. Each level consisted of five biological replicates. (TIF) [file pone.0231741.s003.tif]
